# When light hurts: Comparative Morphometry of Human Brainstem in Traumatic Photalgia

**DOI:** 10.1038/s41598-018-24386-z

**Published:** 2018-04-19

**Authors:** Lora T. Likova, Christopher W. Tyler

**Affiliations:** 1 Smith-Kettlewell Eye Research Institute, 2318 Fillmore Street, San Francisco, 94115 USA; 2 Division of Optometry, School of Health Sciences, City University of London, Northampton Sqaure, London, EC1 0HB UK

## Abstract

Traumatic brain injury is an increasingly common affliction, although many of its serious repercussions are still underappreciated. A frequent consequence is the development of light-induced pain, or **‘**photalgia’, which can often lead to prolonged debilitation. The mechanism underlying the sensitivity to light, however, remains unresolved. Since tissue oedema (swelling) is a common feature of traumatic brain injury, we propose that the brainstem oedema, in particular, might sensitize the brainstem trigeminal complex to signals from ocular mechanisms activated in bright light. To assess this hypothesis, we ran high-resolution Magnetic Resonance Imaging of the brainstems of concussion groups with mild and severe photalgia, without photalgia, and healthy controls. The 3D configuration of the brainstem was determined by Tensor-Based Morphometry (TBM) for each participant. The TBM revealed significant deviations in the brainstem morphology of all concussion groups, with a characteristic signature for each group. In particular, concussion without photalgia showed bilateral expansion at the pontine/medulla junction, whereas concussion with photalgia showed mid-pontine shrinkage, consistent with degeneration of nuclei of the trigeminal complex. These results support the hypothesis that brainstem shrinkage/degeneration represents a morphological substrate of the photalgic sensitization of the trigeminal pathway.

## Introduction

Mild traumatic brain injury (mTBI) is a term generally applied to cases of non-penetrating trauma to the head that results in damage to the brain. As such, it can present a diagnostic and treatment challenge, since the damage is internal to the closed head and is difficult to directly assess, although persistent symptoms may markedly affect the patients’ quality of life.

Light-induced pain or discomfort, or **‘**photalgia’ (otherwise known as light sensitivity, photophobia, asthenopia, photoallodynia or photodynia) is a well-known consequence of mTBI, being commonly associated with decreased tolerance of light levels easily withstood by others^1^, with headaches, pain in the eyes, difficulties in concentration and diminished cognitive function^2^. Photalgia is one of the most common conditions in mTBI patients, occurring in about 60% of blast injury sufferers in the military^3^, and is often debilitating for many years^4^. Further possible ocular pain-inducing sources include, inflammation of the trigeminal nerve pathway, overactive vascular constriction, and deficits of binocular coordination^5–7^, affecting functioning and quality of daily and professional life^8,9^. In unselected civilian groups, about 20% of those with mTBI have photalgia of some kind^10^ and in those who already have a range of visual problems, the proportion increases to about 50%^11^. Given the high rate of mTBI events, these represent large numbers of sufferers in the general population.

The symptoms of photalgia are difficult to treat, in part because its mechanisms are poorly understood. A common link in many of the theories of photalgia is a sensitization of the *trigeminal nerve* to bright light^12–15^, which has also been demonstrated in animal models^16^, although the mechanism by which the trigeminal nerve is sensitized to light remains unresolved. On the other hand, a prevalent feature of mTBI is the *tissue oedema (swelling)* in many deep brain structures^17,18^. Oedema has two basic origins: vasogenic (breakdown of the blood-brain barrier) and cellular (accumulation of fluids inside or outside cell walls)^17,19^. Both origins are associated with mTBI, especially the cellular oedema that results from axonal damage^20–22^. This line of reasoning led us to the hypothesis that the initial oedema might be followed by a chronic shrinkage/degeneration that results in sensitization of the trigeminal nuclear complex to overactivation by light stimulation.

**Figure 1:**
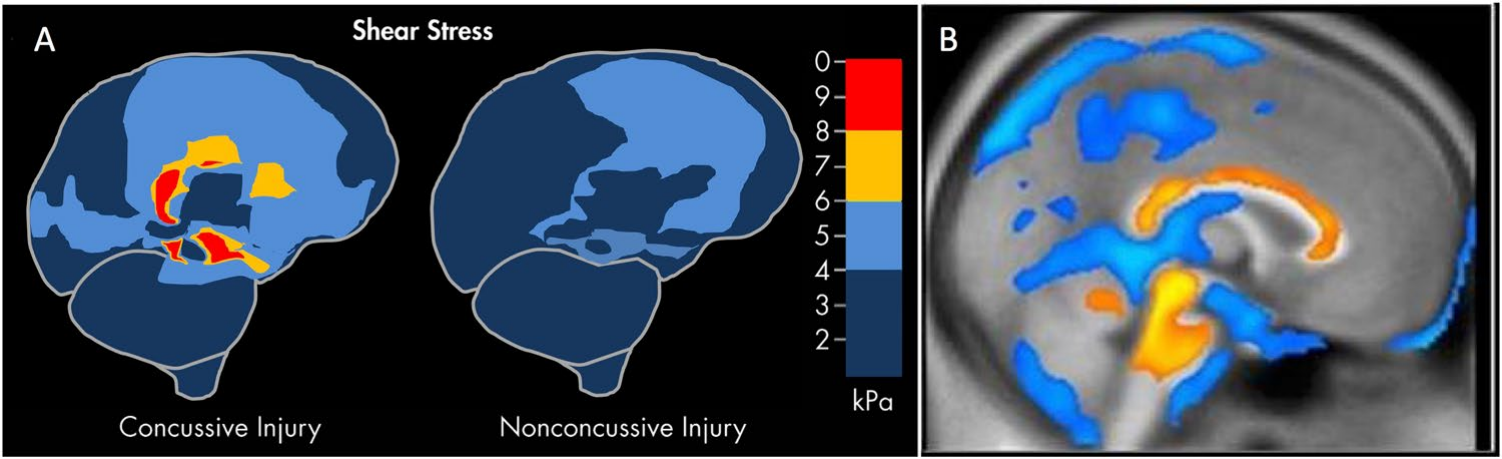
Brainstem focus of concussive effects from two different kinds of published studies. (**A**) Computer modeling of sheer stress forces within the brain following a strong blow to the head. The level and location predicted sheer stresses differentiate concussive (left panel) from nonconcussive (right panel) injuries (based on information from Fig. 1 in Mendez CV, Hurley RA, Lassonde M, Zhang L, Taber KH. Mild traumatic brain injury: Neuroimaging of sports-related concussion. J Neuropsychiatry Clin Neurosci. 17:297–303, 2005). (**B**) Morphometric study of the regions of significant volume reduction (yellow) and increase (blue) in severe TBI between 8 weeks and 12 months post-injury (Reprinted from Sidaros A, Skimminge A, Liptrot MG, Sidaros K, Engberg AW, Herning M, Paulson OB, Jernigan TL, Rostrup E. Long-term global and regional brain volume changes following severe traumatic brain injury: A longitudinal study with clinical correlates. Neuroimage 44(1):1–8, 2009, with permission from Elsevier).

A specific basis of this oedema-based hypothesis for traumatic photalgia is the established result that apparently *diffuse* brain trauma does in fact have a *focal* effect centered on the brainstem^23^ (Fig. 1). The main forms of impact that produced concussion were oblique impacts causing rotational acceleration, which generated focal shear stresses at three main sites in the midbrain and corpus callosum precisely centered on the loci of atrophy following severe TBI found in a morphometric study by Sidaros *et al*.^18^. Note that persistent axonal swelling and disconnection have been observed to continue for years after the initial trauma and are thought to contribute to the development of progressively greater disability in some individuals after TBI^24^. For example, Johnson *et al*.^25^ state, “Increasing evidence suggests that neuroinflammation and microglial activation in the white matter may also contribute to cellular damage^26^ and remarkably, can persist for even years after injury in humans^27,28^.

**Figure 2:**
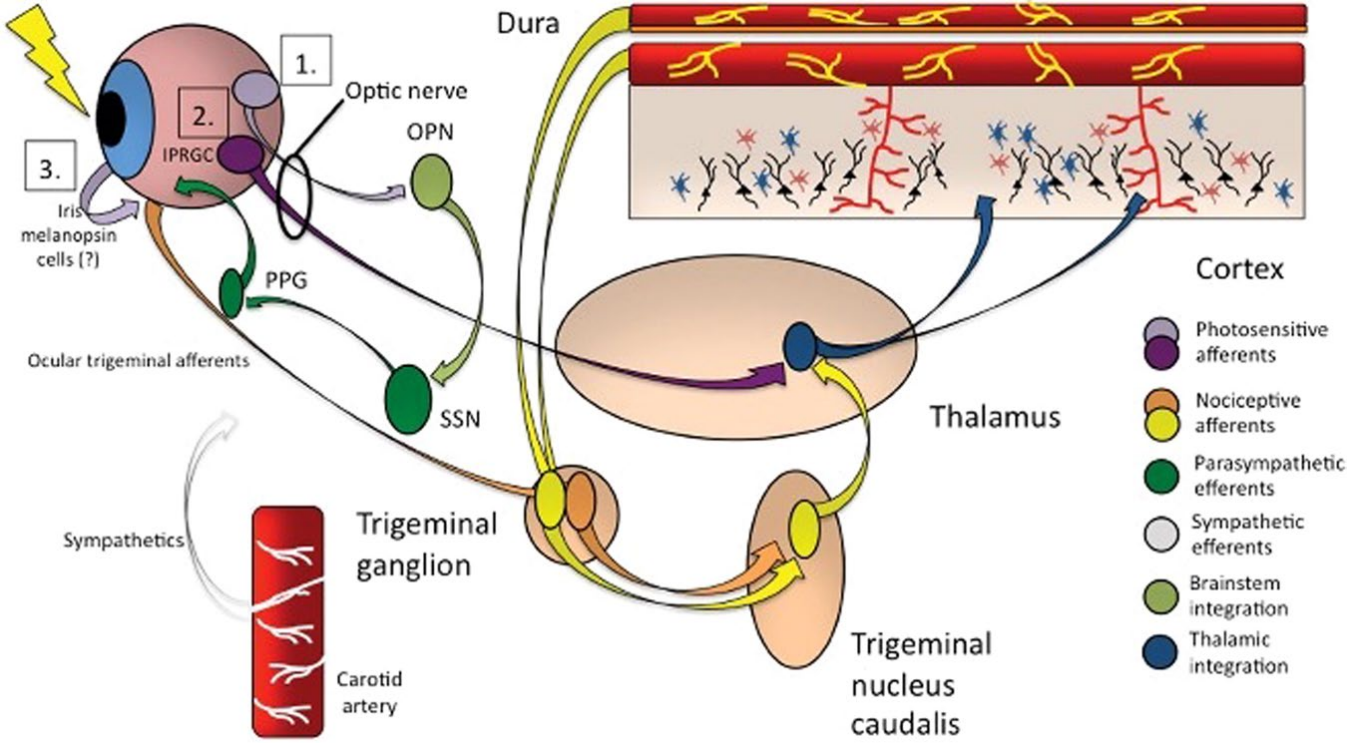
Photalgia circuits. (1) Ganglion cells project light-related signaling to the olivary pretectal nucleus (OPN) that activate superior salivatory nucleus (SSN), which via pterygopalatine ganglion, causes ocular vasodilation and activation of ocular trigeminal afferents (orange) that are heavily expressed on blood vessels. These afferents, with cell bodies in the trigeminal ganglion, project to trigeminal nucleus caudalis, ventroposteromedial thalamus (VPM) and cortex. (2) Intrinsically photosensitive retinal ganglion cells (mRGCs) project directly to thalamic neurons (blue), which also receive intracranial nociceptive afferent signal from neurons in trigeminal ganglion and trigeminal nucleus caudalis of the brainstem (TNC; yellow). (3) VPM neurons, which fire in response to light and pain stimuli, project diffusely to sensory and association cortex (from^12^, with permission; promotional and commercial use of the material in print, digital or mobile device format is prohibited without the permission from the publisher Wolters Kluwer. Please contact healthpermissions@wolterskluwer.com for further information).

Deep brain sites that may be involved in traumatic photalgia (Fig. 2) include the trigeminal nuclear complex (TNC), the periaqueductal gray (PAG), and the ventroposteromedial thalamus (VPM). It is notable that the conjunctiva, cornea, sclera, and uvea (iris, ciliary body, and choroid) are all densely innervated with trigeminal fibers, and thus exquisitely sensitive to pain^29^. Activity in the human TNC can be recorded by standard fMRI techniques to both painless (tactile) and noxious (heat) stimulation, with opposite effects on the BOLD activation of the TNC; activation for the noxious and reduction for the painless stimulation^30^.

Further support for the trigeminal hypothesis has been reported^31^ in a case study of acute ocular photalgia in the left orbit. Significant BOLD activation was found in the left trigeminal ganglion (TG), bilateral TNC, and right VPM during the period of sensitivity, with no significant activation in these regions after recovery. Other brainstem pain loci have been identified as pain modulators during migraine attacks - the adrenergic locus coeruleus, the serotonergic nucleus raphe magnus, and the PAG^32,33^.

## Results

We assessed our brainstem oedema hypothesis by running high-resolution Magnetic Resonance Imaging of the brainstems of three groups of concussion participants – those with mild photalgia (**mild-Ph**), severe photalgia (**severe-Ph**), and without photalgia (**non-Ph**) - and a group of healthy controls (**controls**). The 3D-configuration of each participant’s brainstem was determined by Tensor-Based Morphometry (TBM), as described in Methods.

**Figure 3:**
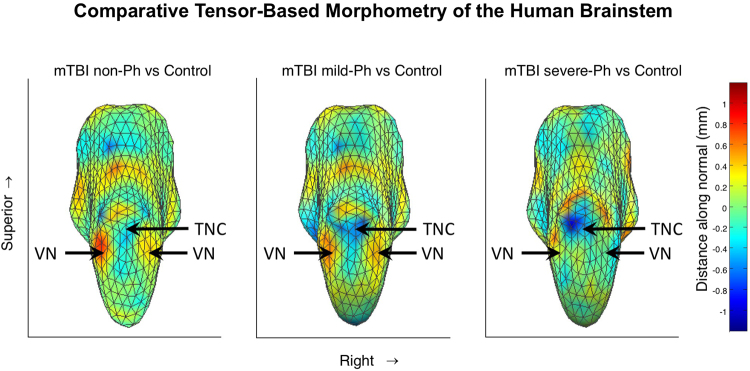
Tensor-based morphometry of the human brainstem. (**A**) mTBI without photalgia shows bilateral expansion at the pontine/medulla junction. (**B**) mTBI with mild photalgia shows local mid-pontine shrinkage, and expansion at the pontine/medulla junction. (**C**) mTBI with severe photalgia shows marked left mid-pontine shrinkage with expansion above it, and much reduced local expansion at the junction with the midbrain. Arrows indicate the peak locations for the statistical assessments. TNC: trigeminal nuclear complex; VN: vestibular nuclei.

**Figure 4:**
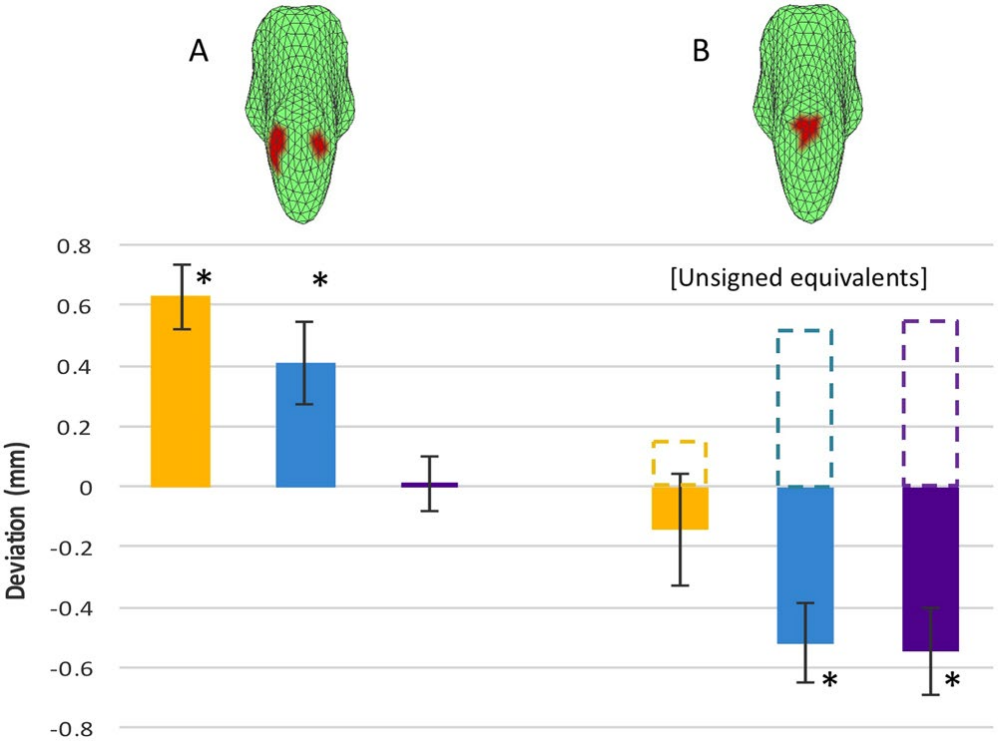
Quantitative TBM analysis. Values of the average deviation for each of three mTBI groups for each of the two masked regions specified by the icons above the bar graphs. (**A**) Positive deviation/*swelling*. (**B**) Negative deviation/*shrinkage*. Note the pattern of symmetrical regions of *swelling* at the pontine/medulla junction and *shrinkage* in the mid-pontine zone. The *dashed* bars in B represent the *absolute* values of the negative deviations, used for the assessment of the *unsigned* interactions between the two zones of interest in Table 1. Non-Ph mTBI: yellow; mild-Ph mTBI: blue; severe-Ph mTBI: purple. Error bars represent one standard error of the means. Asterisks: significant two-tailed at p < 0.05 with the Benjamini-Hochberg correction for false discovery at 0.95.

The changes in the brainstem morphometry for the three experimental mTBI groups relative to the morphometrically registered average brainstem structure for the control group of healthy subjects, are shown in Fig. 3. For non-Ph mTBI, the most prominent effect is a bilateral swelling of the brainstem at the pons/medulla junction and at the level of the pons-midbrain junction (shown in the posterior view of the brainstem; Fig. 3A). In the mild-Ph mTBI group (Fig. 4B), a similar swelling pattern is apparent, with a swath of shrinkage appearing just rostral to these two regions of swelling. In the severe-Ph mTBI group (Fig. 3C), the swelling is much reduced, but it is replaced instead by further shrinkage in the rostral region. There was no significant variation in the morphology of any anterior brainstem structures in any of the groups.

To assess the significance of these morphometric deviations *across* the groups, we first defined the bilateral brainstem zones of greatest deviations in the *intermediate* case, i.e., in the mild-Ph group, starting from the maximum positive and negative values and extending down to a predefined criterion level. This approach defined symmetrical zones of maximal *swelling* at the pontine/medulla junction and *shrinkage* in the mid-pontine in this intermediate case (inset maps in Fig. 4A,B, respectively). We then used these two extrema as *zones of interest* for the comparative statistical analysis, based on both the comparison of the signed deviations across the three photalgia groups in the two zones of interest and the interactions revealed by assessment of the unsigned, or absolute magnitudes of the, deviations from the control brainstem morphology.

Before analyzing the magnitude of the morphometric deviations, we ran a control analysis to determine if there was an effect of time after injury on the results. Because the mTBI symptoms may recover with an approximately exponential function of time since the event^34^, such a relationship might be expected in the present data; the appropriate metric for such an assessment is therefore the correlation of the mean deviation with log time elapsed (which, on the exponential hypothesis, would be a linear function of the average deviation within each ROI in Fig. 4). Neither of these correlations of mean deviation with log time elapsed was significant, however (ROI A: r = 0.02, p = 0.94, n.s.; ROI B: r = −0.17, p = 0.53, n.s.).

In terms of the *signed* - shrinkage/swelling - morphometric deviation, an analysis of variance (Table 1, left panel) in the two *zones of interest* defined above (*cf*. Fig. 4) showed that there are highly significant main effects of *brainstem zone* (mid-pontine, pontine/medulla) and *photalgia level* (none-, mild-, severe-Ph), but no interaction between zone and level. (Based on the choice of *opposite* extrema to define the two zones, the significant effect of brainstem zone is expected, but this factor was included to assess the possibility of an interaction with photalgia level. However, when comparing positive and negative values of a variable, the appropriate measure of strength of an interaction is the *unsigned (absolute*) deviation from the control values). Running a second ANOVA on the *unsigned* magnitudes of the deviations (Table 1, right panel; *cf*. Fig. 4, dashed lines) shows no main effect on either variable, but reveals a highly significant interaction between them that indicates a major functional difference between the two brainstem zones analyzed. The zone defined by maximum *swelling* is most affected in non-Ph mTBI and declines to a negligible level for severe-Ph mTBI, whereas the region defined by maximum *shrinkage* has the opposite behavior: least affected in non-Ph mTBI and most in severe-Ph mTBI.

**Table 1:** Analysis of Variance.

Data Type	Signed Deviation Values					Absolute Deviation from Controls				
Source	SS	df	MS	F	P	SS	df	MS	F	P
Brainstem region	0.91	1	0.91	8.6	**0.007**	0.01	1	0.01	0.09	0.767
Photalgia level	5.15	2	2.58	24.35	**<0.001**	0.17	2	0.8	24.35	0.460
Interaction	0.26	2	0.13	1.23	0.309	1.19	2	5.63	5.633	**0.009**
Error	2.75	26	0.11			2.75	26	0.11		
Total	9.07	31				4.12	31			

**SS**: sums of squares; **df:** degrees of freedom; **MS:** mean squares; **F:** Fisher analysis of variance statistic;

**P**: probability of null hypothesis.

In summary, these two forms of analysis tell us the following:

1. The main effect of *brainstem zone* is equal in strength and opposite in sign (i.e., not significantly different on the basis of the *unsigned* ANOVA, but significantly opposite – swelling (4A) vs shrinkage (4B) – on the basis of the *signed* ANOVA).
2. The main effect of *photalgia level* is equal in strength for the swelling and shrinkage (i.e., not significantly different on the *unsigned* ANOVA).
3. Photalgia level has an inverse effect on the absolute size of the morphometric deviation in the two zones (i.e., the *unsigned* interaction is significant).

Similarly, a Benjamini-Hochberg post-hoc analysis^35^ indicates that four of the individual brainstem zone comparisons show significant deviations from the control brainstem morphology (asterisks in Fig. 4); the non-Ph and mild mTBI groups show significant *swelling* in the pontine/medulla junction region and the mild-Ph and severe-Ph mTBI show significant *shrinkage* in the mid-pontine region.

## Discussion

The TBM analysis revealed significant deviations in the brainstem morphology of all groups with traumatic brain injury. Furthermore, these deviations were *characteristic* for each group. First, there was a pronounced difference between the patterns of average swelling and shrinkage as a function of the severity of the photalgia. In the non- and mild-photalgic mTBI groups, the predominant effect was a bilateral swelling at pontine/medulla junction, in the region of the vestibular nuclei and the underlying facial nuclei of the TNC. In the mild- and severe-photalgic mTBI groups, the most significant effect was shrinkage of the brainstem with a maximum toward the posterior center of the pons, in the general location of the trigeminal sensory nuclei of the TNC. The finding of shrinkage in this critical zone of the brainstem in the photalgia groups suggests that it may be caused by local degeneration of the trigeminal pathway as a result of the mTBI events. The swelling in the region of the vestibular/facial nuclei is somewhat harder to understand, but may represent chronic oedema that developed in this region.

This analysis is offered with the recognition that the brainstem consists of a large array of nuclei and specialized pathways, and that any pattern of variation in surface morphology could arise from multiple combinations of changes through this three-dimensional array, so that a firm identification with any given brainstem nucleus cannot be expected in a human morphometric study. Nevertheless, it is remarkable that the changes are occurring in regions that can be identified with aspects of the trigeminal pathway, which is the main carrier of pain signals from the facial region by way of the 5^th^ cranial nerve. The present results therefore provide supportive evidence for the hypothesis proposed here, that this pathway plays a role in the manifestations of photalgia, whose specific mechanism is otherwise unresolved.

## Methods

### Participants

The participants were recruited from a non-academic population via a social media website. This non-standard recruitment strategy was adopted as a form of outreach to the broader population with long-term sequelae to mTBImTBI, which is generally inaccessible to recruitment among recent hospital discharges. All recruitment and experimental procedures adhered to the Declaration of Helsinki. The experimental protocol was approved by the Smith-Kettlewell Institutional Review Board; prior to participating, all volunteers provided their informed consent.

### Inclusion Criteria

The participants were included in the analysis if they met the criteria of letter acuity of 20/40 or better in both eyes (Bailey-Lovie chart, mean LE denominator: 22 ± 5, mean RE denominator: 23 ± 6), of having no reportable ocular abnormalities, and of having had mTBI events defined as involving closed-head trauma resulting in a loss of consciousness for a period of 5 minutes or more, or loss of memory of the traumatic event *per se*.

### Exclusion Criteria

Infants and children were excluded because of the need for homogeneous comparison across the groups. As a standard exclusion criterion at the neuroimaging center, pregnant women were excluded to avoid any possible risk to the fetus from fMRI scanning. Non-fluent English speakers were excluded because of the need for the subject to follow the experimental instructions. Participants with a medical history of brain disease, including epilepsy, and those taking prescription or illicit drugs were excluded to avoid confounding the effects with those of brain trauma.

### Participant Characteristics

Four groups of participants were tested: (i) a group who had experienced mTBI but with no resulting photalgia or light-induced pain symptoms, (ii) a mTBI group with mild photalgia symptoms, (iii) a mTBI group with severe photalgia, and (iv) a healthy, non-TBI control group. The 16 mTBI participants had an average age of 60 years (64% male) while the five controls averaged 56 years (80% male). Five mTBI participants were non-Ph, seven were mild-Ph, and four were severe-Ph. Specifics of participant characteristics are listed in Table 2.

**Table 2:** Participant Characteristics.

Condition	ID	Gender	Age	Years since conc.	Number of conc.	Duration of conc.	Photalgia status	Neurological deficits	Medical issues	Stereo test	Memory Problem
mTBI	MRGC001	M	60	0.05	1	0.05	Mild Ph	Headache		2	2
	MRGC002	M	81	3	1	unk	Mild Ph	Balance	Stroke	2	2
	MRGC003	M	58	6	1	1	Mild Ph	Insomnia		2	0
	MRGC004	M	82	4	2	20	Severe Ph	Balance	Cataract	2	2
	MRGC005	M	66	40	1	unk	non-Ph			2	0
	MRGC007	M	65	0.1	1	0.08	non-Ph		Heart	2	2
	MRGC008	F	60	0.25	1	unk	Severe Ph			2	2
	MRGC009	M	55	0.9	2	0.5	Mild Ph	Insomnia		2	0
	MRGC012	M	48	3	1	168	non-Ph			2	2
	MRGC013	M	57	3	1	72	non-Ph	Insomnia	Asthma	2	0
	MRGC014	M	58	5	5	0.5	Severe Ph	Balance	HIV	2	0
	MRGC015	F	68	8	1	0.133	Mild Ph	Headache		2	0
	MRGC016	M	64	44	3	0.05	non-Ph			2	0
	MRGC018	F	53	0.35	2	0.033	Mild Ph	Headache		2	0
	MRGC019	F	42	19	2	4	Severe Ph	Insomnia		2	0
	MRGC020	F	43	12	2	0.08	Mild Ph	Headache		2	2
	**%M**	**64**	**60**	**2.59**	**1.69**	**0.73**					
Control	MRGC021	M	66							2	0
	MRGC022	M	39							2	0
	MRGC023	F	59							2	0
	MRGC024	M	70							2	0
	MRGC025	M	38							2	0
	**%M**	**80**	**56**	**0**	**0**	**0**					

**M** - male, **F** - female, **Years since conc**. - years since concussion, **Number of conc**. - number of concussions, **Duration of conc. -** duration of concussion in hours, **unk** - unknown, **Stereo test -** 2 - normal, 1 - some loss, 0 - no stereo, **Memory problem** - 2 - yes, 1 - between no and yes, 0 - no problems, **Ph** - photalgia.

The mTBI participants underwent three forms of assessment, a *medical history* for neurological deficits, general medical issues and memory issues, a 1 arcmin *random-dot stereo test*, and *a behavioral test* assessing their sensitivity to light^36,37^. As with all medical histories, there is a risk of incomplete recall of details, however, these are not relevant for the group categorization of the participants in this study, which is based on the behavioral measurements of the level of photophobia. The behavioral task consisted of a nociphysical ascending staircase procedure of setting the ocular pain threshold for a full 180° white field stimulus flickering with a 2.5 Hz square wave at 100% contrast. Participants were assigned to the severe-Ph group if their nociophysical threshold for this stimulus was below 50 cd/m^2^ the mild-Ph grou*p* if the threshold setting was between 50 cd/m^2^ and the maximum stimulus intensity of 265 cd/m^2^, and to the non-Ph group if they were comfortable with the maximum intensity.

### MRI Methods

An MPRAGE protocol on a 3 T Siemens Trio with repetition, echo, inversion times of 2300, 2.96 and 900 s and a flip angle of 9° with 0.8 × 0.8 × 0.8 mm voxels was used. A few scans had minor variations from this protocol due to equipment upgrades that should have had no effect on the morphometric parameters. The T1 volume was processed with the FIRST tools from FSL (Analysis Group, FMRIB, Oxford, UK), which provided an affine registration to a standard brain metric (the MNI152; Montreal Neurological Institute, Montreal, Canada), and generated a triangular mesh representation of the brainstem. The 3D coordinates of each brainstem vertex were transformed to the standard space with this affine registration to reduce the overall size and the x, y, z aspect ratio variations among participants in order to focus on localized deformities that may be due to mTBI. The average vertex locations across all participants in both the control and test groups was then computed, and each individual participant was further registered in MNI152 space to the group average using a higher order linear transform that included all 3rd-order and lower terms of the 3-dimensional vertex coordinate array.

Next, the average vertex locations of the three mTBI groups were computed as the difference between each of them and the healthy control group coordinates and projected onto an outward-facing surface normal to the difference tensor representing the comparative distortion of the mTBI group brainstems. Data were visualized as a rendering of the surface of the control group coloured by the average magnitude of the difference tensor at each vertex, for each of the mTBI groups.
